# AMPK Signaling Regulates Mitophagy and Mitochondrial ATP Production in Human Trophoblast Cell Line BeWo

**DOI:** 10.31083/j.fbl2704118

**Published:** 2022-04-01

**Authors:** Bin Wu, Yun Chen, Robert Clarke, Emmanuel Akala, Peixin Yang, Bin He, Haijun Gao

**Affiliations:** 1 Department of Reproductive Medicine, Central Hospital Affiliated to Shandong First Medical University, 250013 Jinan, Shandong, China; 2 Rocket Pharmaceuticals, Inc., Cranbury, NJ 08512, USA; 3 The Hormel Institute, University of Minnesota, Austin, MN 55912, USA; 4 Department of Pharmaceutical Sciences, College of Pharmacy, Howard University, Washington, DC 20060, USA; 5 Department of Obstetrics, Gynecology and Reproductive Sciences, University of Maryland School of Medicine, Baltimore, MD 21201, USA; 6 Reproductive Physiology Laboratory, National Research Institute for Family Planning, 100081 Beijing, China; 7 Department of Physiology and Biophysics, College of Medicine, Howard University, Washington, DC 20059, USA

**Keywords:** AMPK, mitophagy, mitochondria, trophoblast, BeWo, ATP production, human

## Abstract

**Introduction:**

Accumulating evidence suggests that mitochondrial structural and functional defects are present in human placentas affected by pregnancy related disorders, but mitophagy pathways in human trophoblast cells/placental tissues have not been investigated.

**Methods:**

In this study, we investigated three major mitophagy pathways mediated by PRKN, FUNDC1, and BNIP3/BNIP3L in response to AMPK activation by AICAR and knockdown of *PRKAA1/2* (AKD) in human trophoblast cell line BeWo and the effect of AKD on mitochondrial membrane potential and ATP production.

**Results:**

Autophagy flux assay demonstrated that AMPK signaling activation stimulates autophagy, evidenced increased LC3II and SQSTM1 protein abundance in the whole cell lysates and mitochondrial fractions, and mitophagy flux assay demonstrated that the activation of AMPK signaling stimulates mitophagy via PRKN and FUNDC1 mediated but not BNIP3/BNIP3L mediated pathways. The stimulatory regulation of AMPK signaling on mitophagy was confirmed by AKD which reduced the abundance of LC3II, SQSTM1, PRKN, and FUNDC1 proteins, but increased the abundance of BNIP3/BNIP3L proteins. Coincidently, AKD resulted in elevated mitochondrial membrane potential and reduced mitochondrial ATP production, compared to control BeWo cells.

**Conclusions:**

In summary, AMPK signaling stimulates mitophagy in human trophoblast cells via PRKN and FUNDC1 mediated mitophagy pathways and AMPK regulated mitophagy contributes to the maintenance of mitochondrial membrane potential and mitochondrial ATP production.

## 1. Introduction

The placenta plays a critical role during pregnancy by maintaining pregnancy, nurturing the fetus and mediating bidirectional communication between the mother and the fetus. More importantly, the placenta is a key mediator of fetal programming by which the long term health and disease risk of offspring is predisposed by the *in utero* environment such as nutrition, inflammation, endocrine status [2-4]. Placental functions require a high demand of energy. Thus, the placenta is an active metabolic tissue, accounting for 40 percent of oxygen consumption [5] and one third of glucose uptake by the placental-fetal unit during late pregnancy [6]. Like other metabolic tissues, mitochondria in the placenta provide the majority of ATP production by metabolizing three major energy substrates: glucose, lipid, and amino acids [7,8]. In addition to the central role in cellular energy metabolism, mitochondria regulate a variety of biochemical events such as calcium signaling, reactive oxygen species production, apoptosis and steroidogenesis [9,10]. Therefore, mitochondrial quality and quantity control is indispensable for mitochondrial functions and consequent cellular functions.

Mitophagy, a highly conserved and specific process to remove the dysfunctional/destroyed mitochondria, in concert with mitochondrial biogenesis, is critical for maintaining mitochondrial homeostasis and functions [11]. Mitophagy pathways have been studied primarily in cardiac and neuronal cells due to the high demand of mitochondrial ATP production in the heart and neuronal tissues [12,13]. Currently, mitohpagy is thought to proceed as follows. Depolarization of mitochondrial membrane or other impairment interrupts normal proteolytic processing of PINK1 kinase. As a consequence, PINK1 accumulates on the outer mitochondrial membrane and phosphorylates MFN2, promoting the recruitment of PRKN and PRKN mediated ubiquitinization of proteins on the outer mitochindrial membrane. Poly-ubiquitinated proteins bind to SQSTM1 protein, which is recognized and bound to LC3 protein in the autophagosome, leading to engulfing mitochondrial proteins into autophagosomes. Following the fusion of autophagosome and lysosome, mitochondrial proteins are degraded by lysosomal proteases [11]. To date, in mammals, three major mitophagy pathways have been defined primarily by the key mediator proteins in mitochondrial or mitochondrial fragmentation recognition and engulfing in each pathway, PINK1-PRKN (mitophagy receptor independent pathway), BNIP3-BNIP3L, and FUNDC1 (mitophagy receptor dependent pathways), respectively [14,15]. These mediators demonstrate a preferential association with LC3 family members in recruiting autophagosomes that encapsulate mitochondria [16]. These mitophagy pathways are responsive to different cellular stimuli or stress. Briefly, PINK1-PRKN mediated mitophagy responds to mitochondrial membrane potential depletion affected by stresses, such as oxidative stress; BNIP3- BNIP3L mediated mitophagy responds to nutritional stress and/or stimuli [13] and hypoxia [17]; FUNDC1 mediated mitophagy responds to mitochondrial uncoupling, and hypoxia [18,19].

It has been widely accepted that mitophagy is a selective form of autophagy by which the cell recycles macromolecules and organelles to maintain cell survival and thus, sharing similar regulatory mechanisms to autophagy [20,21]. AMPK signaling and mTOR signaling are major counterregulatory mechanisms of the formation of autophagosomes, a critical regulatory procedure in the initiation of autophagy and subsequent events [22-25]. AMPK signaling stimulates the initiation of autophagy by AMPK-ULK1 axis in response to nutritional deprivation, while mTOR signaling inhibits the initiation of autophagy [24]. Accumulating evidence suggests that AMPK signaling regulates autophagy in the placenta and/or trophoblast cells, and its regulatory role in autophagy may be disrupted by major pregnancy related disorders. AMPK activation reflects its phosphorylation at Thr172 and was reduced in the placentas from women who were obese prior to their pregnancy [26,27], gestational diabetes mellitus (GDM) [28], preeclampsia [29] and preterm birth [30], compared their counterparts with normal pregnancy. In addition, mitochondrial structural and functional defects have been found in the placentas from women with maternal obesity and GDM [28,31-34]. These observations suggest that a causal relationship exists between AMPK signaling and autophagy/mitophagy in human placentas and/or trophoblast cells.

In this study, we hypothesized that AMPK signaling stimulates mitophagy in human trophoblast cells. To test this hypothesis, first, using human trophoblast cell line, BeWo, a widely applied human trophoblast cell model, we investigated the effect of AMPK activator AICAR (5-Aminoimidazole-4-carboxamide ribonucleotide) on the following parameters, the phosphorylation of AMPK and its target proteins ACC and ULK1, autophagy mediators LC3II, SQSTM1, and mitochondrial receptors PRKN, BNIP3/BNIP3L, and FUNDC1 in total cell lysates and/or mitochondria-enriched fractions, and mitochondrial membrane potential. Second, to confirm the role of AMPK signaling in mitochondrial mitophagy in human trophoblast cell, we investigated mitophagy pathways, mitochondrial membrane potential, and mitochondrial ATP production in response to AMPK knockdown. Our study showed that AICAR activated AMPK signaling stimulates mitophagy in BeWo cells via PRKN and FUNDC1 mediated processes, while AMPK knockdown inhibited mitophagy, increased mitochondrial membrane potential, and reduced mitochondrial ATP production.

## 2. Materials and Methods

### 2.1 Cell Line and Cell Culture

Human cytotrophoblast cell line, BeWo (Cat. CCL-98; ATCC, Manassas, VA, USA) was cultured in F-12K culture medium (Cat. 30-2004; ATCC) supplemented 10% FBS (Cat. 30-2020; ATCC) and penicillin/streptomycin (Cat. 30-2300; ATCC) in a 5% CO_2_ atmosphere at 37 °C. In AICAR stimulated autophagy/mitophagy flux assay, BeWo cells were cultured in 60-mm culture dishes at the density of 2.5 million cell per dish. After cells attached to the plate overnight, cells were treated with AICAR (0.5 *μ*M; Cat. 10010241; Cayman Chemical, Ann Arbor, MI, USA), Chloroquine (40 *μ*M; Cat. 14194; Cayman Chemical) or their combinations for 24 hours. In PRKAA1/2 knockdown (AKD) induced autophagy/mitophagy flux assay, AKD and BeWo cells were treated with Chloroquine for 24 hours, and those without treatment served as controls. The cell culture information for mitochondrial membrane potential and ATP production assays were described in each section below.

### 2.2 AMPK Knockdown in BeWo Cells

Using AMPK lentivirus shRNA targeting both alpha 1 and 2 subunits PRKAA1/2 (Cat. No. sc-45312-V; Santa Cruz Biotechnology, Dallas, TX, USA), AMPK knockdown was conducted according to the manufacturer’ instructions. Briefly, BeWo cells seeded in 24-well cell culture plate was transfected by shRNA Lentiviral Particles for 36 hours, followed by cell recovery, proliferation, and puromycin selection for 6 days. The AMPK knockdown was confirmed by reduction in both mRNA and protein levels, measured by q-PCR and Western blotting analysis, respectively. The gene knockdown was stable in as least 9 passages as we have determined recently.

Total mRNAs extraction and on-column genomic DNA cleanup were conducted using PureLink^™^ RNA Micro Scale Kit (Cat. 12183016; Invitrogen, Waltham, MA, USA) and all procedures were followed the manufacturer’s instructions. cDNAs were made from 1 *μ*g of totals RNAs using iScript^™^ cDNA Synthesis Kit (Cat. 1708890; Bio-Rad, Hercules, CA, USA). The mRNA levels of genes, PRKAA1/2, were measured by q-PCR that was performed on a CFX Connect Real-Time PCR Detection System (Cat. 1855200; Bio-Rad), using iTaq™ Universal Probes Supermix (Cat. 1725135; Bio-Rad). The reaction mixture was incubated at 95 °C for 10 min and cycled according to the following parameters: 95 °C for 30 seconds and 60 °C for 1 min for a total of 40 cycles. Negative control without cDNA was performed to test primer specificity. The relative gene expression was calculated by use of the threshold cycle (CT) of RNA18S1/CT of PRKAA1/2. The primer sequences and product information are as follows. RNA18S: Forward: CGCCGCTAGAGGTGAAATTCT; Reverse: CGAACCTCCGACTTTCGTTCT; Product size: 101 bp; PRKAA1 Forward: ACAGCCGAGAAGCA-GAAACA; Reverse: CTTCACTTTGCCGAAGGTGC; Product size: 93 bp; PRKAA2: Forward: CTGCTGGCT-TACACAGACCA; Reverse: AGGCGAGGTGAAACT-GAAGA; Product size: 121 bp. All procedures in RNA extraction, cDNA preparation and real-time PCR were conducted according to the manufacturers’ instructions. The procedures for Western blotting were same as described below.

### 2.3 Total Cell Lysates and Mitochondria-Enriched Components Preparation

Total cell lysates and mitochondria-enriched components were prepared, following the procedures described previously [35,36] with minor modifications. Briefly, after culture media was removed, cells were washed with phosphate buffered saline (PBS), lysed in mitochondrial homogenization buffer (10 mM Tris, 1 mM EDTA, and 250 mM sucrose, pH 7.4 and supplemented with protease and phosphatase inhibitor Cocktail (PPC1010; Sigma) and frozen at −80 °C until further protein extraction. Cells were lysed using ultrasonic homogenizer sonicator (Boshi Electronic Technology, Guangzhou, China) with 1% output and 6 cycles of 2-second sonication. Cell lysates were centrifuged at 1500 × g for 10 min 4 °C, then the supernatant was saved as whole cell lysates and partial lysates were centrifuged at 12,000 × g for 15 min to pellet the mitochondria, which was washed in homogenization buffer twice to remove cytoplasm contamination and resuspended in fresh homogenization buffer for further protein concentration estimation and Western blotting analysis.

### 2.4 Western Blotting Analysis

Protein concentration in whole cell lysates and mitochondria enriched components was determined using Pierce^™^ BCA Protein Assay Kit (Cat. 23227; Thermo Fisher Scientific, Waltham, MA, USA) and NanoDrop^™^ One/OneC Microvolume UV-Vis Spectrophotometer (Cat. ND-ONEC-W; ThermoFisher). Twenty microgram of whole cell lysates or mitochondrial proteins was mixed with 4× Laemmli Sample Buffer (Cat. 1610747; Biorad) and heated at 95 °C for 5 min, followed by electrophoresis in 4–20% Criterion^™^ TGX^™^ Precast Midi or Mini Protein Gel (Cat. 5671095, 4561096; Bio-Rad) and membrane transfer using Trans-Blot Turbo Transfer System (Cat. 1704150; Bio-Rad) and RTA Midi or Mini 0.45 *μ*m LF PVDF Transfer Kit (Cat. 1704275, 1704274; Bio-Rad). MemCode staining was conducted on the freshly made blots using MemCode^™^ Reversible Protein Stain Kit (Cat. No. 24585; Thermo Fisher Scientific) and imaged by iBright FL1000 Imaging Systems (Thermo Fisher Scientific). The blot was then blocked in Intercept^®^ (TBS) Blocking Buffer (Cat. 927-60001; LI-COR Biosciences, Lincoln, NE, USA) and incubated with primary antibodies (Table 1) at 4 °C overnight and species matched secondary antibodies at room temperature for 1 hour, then immersed in ECL Western Blotting Substrate (Cat. No. PI32106; Thermo Fisher Scientific) and imaged by iBright FL1000 Imaging Systems. Blot stripping with Restore^™^ PLUS Western Blot Stripping Buffer (Cat. 46430; Thermo Fisher Scientific) and re-blocking were performed when reusing the blot. All procedures in protein electrophoresis, membrane transfer, MemCode Staining, and blot stripping were conducted following the manufacturers’ instructions. At the end, the densitometry of bands for a given target protein in the image was quantified by ImageJ software (NIH) and normalized to that of total proteins in each lane after MemCode staining (for proteins in autophagy/mitophagy flux assay), or that of ACTB (for total and phosphorylated AMPK, ACC and ULK1 proteins).

### 2.5 Measurement of Mitochondrial Membrane Potential

Mitochondrial membrane potential was measured using Tetramethylrhodamine, Methyl Ester (TMRM) staining. Briefly, cells were seeded into 96-well plates at a density of 40,000 cells per well, attached to the plate overnight and treated with or without Chloroquine (40 *μ*M) for 24 hours. Cells were then stained with TMRM (250 nM; Cat. No. T668; Thermo Fisher Scientific) and Hoechst 33342 (10 *μ*g/mL; Cat. No. H21492; Thermo Fisher Scientific) for 30 min, and the staining of TMRM and Hoechst 33342 were scanned in red and blue fluorescence channels, respectively, using Celigo Imaging Cytometer (Nexcelom Bioscience, Lawrence, MA, USA) followed by quantification with Expression Analysis Program of Celigo Imaging Cytometer. All procedures in plate setup, scanning and analysis were conducted according to the manufacturer’s standard protocols. In data analysis, cells were segmentated in blue fluorescence channel, and the area defined by the nucleus and proper dilation radius will be used as mask to quantify the total red signals in red fluorescence channel. Mitochondrial membrane potential was presented as the mean intensity of red fluorescence which is the ratio of total fluorescence to the whole cell areas in each well.

### 2.6 Seahorse Cell Mito Stress Test

Seahorse Cell mito stress test is a golden standard assay on mitochondrial ATP production in live cells by measuring oxygen consumption rate in the presence of electron transport chain complex inhibitors (oligomycin, rotenone/antimycin) or ATPase uncoupler Carbonyl cyanide-p-trifluoromethoxyphenylhydrazone (FCCP). AKD and control BeWo cells were seeded into Seahorse Miniplate at a density of 20,000 cells per well (n = 3) and cultured in cell culture incubator with 5% CO_2_ overnight. All cell mito stress test procedures were conducted according to the manufacturer’s optimized protocol, except that Oligomycin A (Cat. 75351; MilliporeSigma, St. Louis, MO, USA) at the concentration of 2 *μ*M and FCCP (Cat. C2920; MilliporeSigma) at the concentration of 0.3 *μ*M were applied to uncouple ATP production in BeWo cells.

### 2.7 Statistics

Data on gene expression and protein abundance were analyzed for the effect of AICAR, Chloroquine and/or their interactions, and data on gene expression and protein abundance, mitochondrial membrane potential and ATP production were analyzed for the effect of AMPK knockdown, using least-squares analysis of variance (ANOVA) and the general linear model procedures of the Statistical Analysis System (Version 9.4., SAS Institute, Cary, NC, USA). Log transformation of variables was performed when the variance of data was not homogenous among groups, as assessed by the Levene’s test. A *p*-value ≤ 0.05 was considered significant. A *p*-value ≥ 0.05 but ≤0.1 was considered a trend to be different. Data were presented as least-squares means (LSMs) with overall standard errors (SE).

## 3. Results

### 3.1 AICAR Stimulates AMPK Signaling

**Fig. 1.**
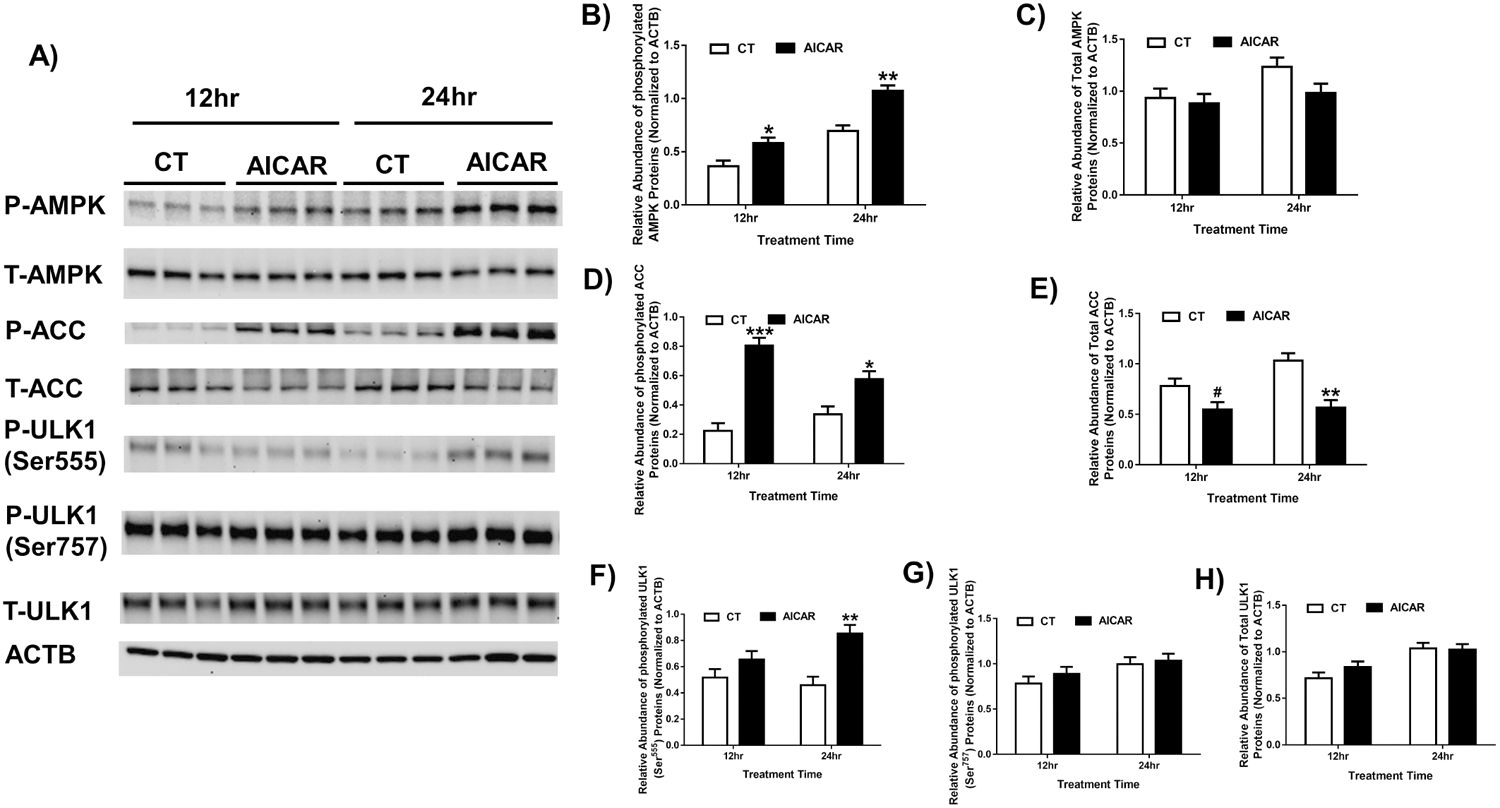
AICAR activated AMPK signaling in human trophoblast cell line BeWo. (A) Western blotting analysis of phosphorylated and total AMPK, ACC, ULK1 after AICAR treatment for 12 or 24 hours. Relative abundance of phosphorylated AMPK at Thr172 proteins (B), total AMPK (C), phosphorylated ACC at Ser79 (D), total ACC (E), phosphorylated ULK1 at Ser555 (F), at Ser757 (G) and total ULK1 (H), all normalized to ACTB. Data are presented as the mean ± SEM (n = 3); *p < 0.05; **p < 0.0E ***p < 0.001.

To confirm that AICAR can stimulate AMPK signaling in human trophoblast cells, we treated BeWo cells with AICAR for 12 or 24 hours and measured the phosphorylation of amino acids residue specific to AMPK activity and immediate downstream target proteins ACC and ULK1. The phosphorylated AMPK at Thr172 (p-AMPK) levels were increased (*p* < 0.05) by 1.59- and 1.54-fold by AICAR after 12 and 24 hours’ treatment, respectively, compared to the control group, while total AMPK protein levels were unchanged (Fig. 1A). Coincidently, the p-ACC (Ser79) levels were increased by 3.61- (*p* < 0.001) and 1.72- (*p* < 0.05) fold by AICAR, respectively after 12 and 24 hours’ treatment, while total ACC protein levels were reduced by 1.43- (0.05 < *p* < 0.1) and 1.82- (*p* < 0.01) fold, respectively (Fig. 1B). p-ULK1 expression (Ser555, involved in initiation of autophagy by AMPK) was increased (*p* < 0.01) by 1.86-fold by AICAR after 24 hours’ treatment (Fig. 1), while p-ULK1 (Ser757, not involved in initiation of autophagy) and total ULK1 protein levels were unaffected by AICAR after 12 and 24 hours treatment. These data indicate that AICAR can stimulate AMPK signaling and the initiation of autophagy/mitophagy is mediated by ULK1 in human trophoblast cells (Fig. 1C).

### 3.2 AICAR Stimulates Autophagy Flux as Well as Mitophagy Flux

Autophagy flux with specific blockage of lysosomal degradation of autophagy target protein is used in autophagy analysis. Chloroquine is one of widely used chemical which inhibits the fusion of autophagosome and lysosome [37]. There is no report on autophagy/mitophagy flux in human trophoblast cells, but the dose of chloroquine is critical in the mitophagy flux analysis due to its cytotoxicity at high doses, so we optimized the dose of chloroquine by investigating the main autophagy/mitophagy mediator LC3II in mitochondria enriched fractions to in response to different doses of CLQ. Our data indicated that CLQ at the concentration of 40 *μ*M sufficiently blocked autophagy and mitophagy flux and did not understate the effect of AICAR (Supplementary Fig. 1). Thus, the combination of 0.5 mM AICAR and 40 *μ*M CLQ was suitable in analysis of autophagy and mitophagy flux in BeWo and thus, being applied in experiments in our study.

**Fig. 2.**
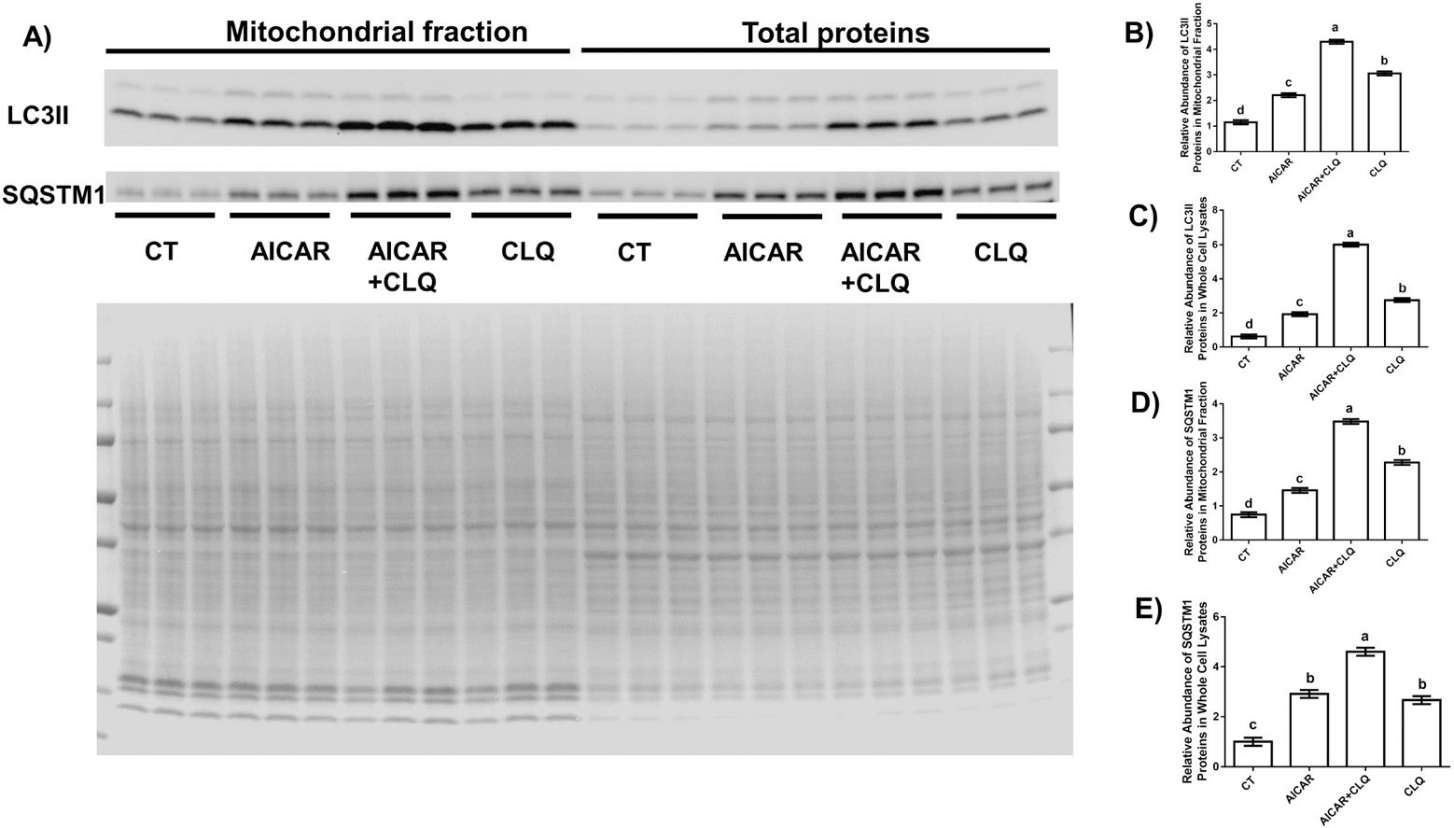
AICAR stimulated autophagy flux in human trophoblast cell line BeWo. (A) Western blotting analysis of LC3II and SQSTM1 in mitochondrial fractions and whole cell lysates extracted from BeWo cells treated with AICAR, CLQ, their combination (AICAR+CLQ) and controls (CT) and MemCode staining. Quantification of the relative abundance of LC3II and SQSTM1 in mitochondrial fractions (B,D) and whole cell lysates (C,E) proteins by normalizing density of a band in Western blot to MemCode staining signal in the entire lane. Data are presented as the mean ± SEM (n = 3); different letters represent statistically significant difference.

In whole cell lysates, AICAR alone increased the protein abundance of LC3II for 3.09-fold (*p* < 0.0001) compared to the control cells, while the combination of AICAR and CLQ further increased the abundance of LC3II proteins by 3.11-fold (*p* < 0.0001) (Fig. 2C). Similarly, AICAR alone increased the protein abundance of SQSTM1 for 2.92-fold (*p* < 0.0001) compared to the control cells, while the combination of AICAR and CLQ further increased the abundance of SQSTM1 proteins by 1.58-fold (*p* < 0.0001) (Fig. 2D).

In mitochondrial fractions, AICAR alone increased the protein abundance of LC3II for 1.92-fold (*p* < 0.0001) compared to the control cells, while the combination of AICAR and CLQ further increased the protein levels of LC3II by 1.95-fold (*p* < 0.0001) (Fig. 2E). Similarly, AICAR alone increased the protein abundance of SQSTM1 for 1.96-fold (*p* < 0.05) compared to the control cells, while the combination of AICAR and CLQ further increased the abundance of SQSTM1 proteins by 2.38-fold (*p* < 0.0001) (Fig. 2F).

### 3.3 AICAR Stimulates the Accumulation of PRKN and FUNDC1 Proteins, but Reduces BNIP3/3L Proteins in Mitochondrial Fractions in BeWo Cells

**Fig. 3.**
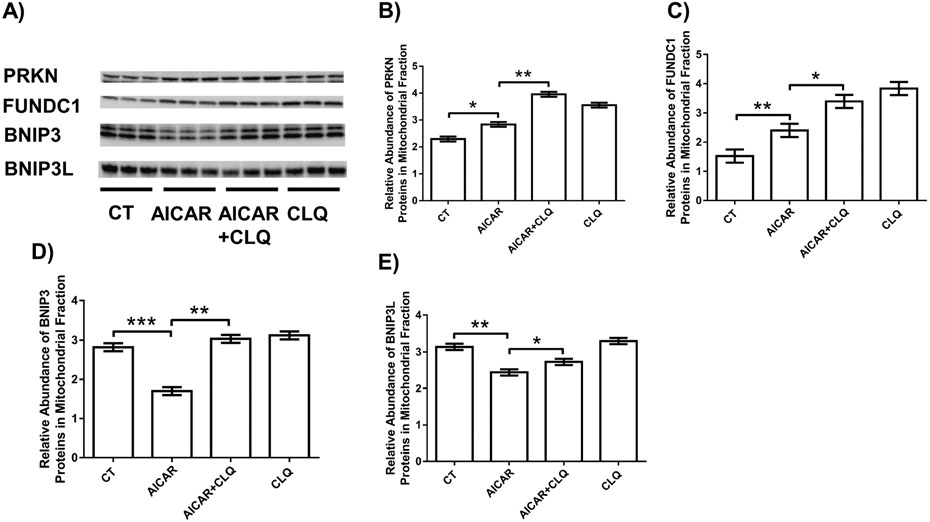
AICAR regulated mitophagy pathways human trophoblast cell line BeWo. (A) Western blotting analysis of on PRKN, FUNDC1, BNIP3, and BNIP3L in mitochondrial fractions from BeWo cells treated with AICAR, CLQ, their combination (AICAR+CLQ) and controls (CT). Quantification of the relative abundance of PRKN (B), FUNDC1 (C), BNIP3 (D) and BNIP3L (E) proteins in mitochondrial fractions by normalizing density of a band in Western blot to MemCode staining signal in the entire lane (shown in Fig. 2). Data are presented as the mean ± SEM (n = 3); * *p* < 0.05; ** *p* < 0.01; *** *p* < 0.001.

We next analyzed mitophagy flux by measuring the abundance of mediators of 3 major mitophagy pathways by Western blotting (Fig. 3A). In mitochondrial fractions, AICAR alone increased the protein abundance of PRKN, and FUNDC1 proteins by 1.24-, 1.58-fold (both *p* < 0.05), respectively, and increased abundance further by the combination of AICAR and CLQ (*p* < 0.05) by 1.39-, 1.38-fold (Fig. 3B,C). In contrast, the abundance of BNIP3 and BNIP3L was reduced by 1.66- and 1.29-fold (both *p* < 0.05) by AICAR, compared to controls, respectively, but increased by 1.78-, 1.12-fold (both *p* < 0.05) by the combination of AICAR and CLQ (Fig. 3D,E).

### 3.4 Knockdown ofPRKAA1/2 Expression Impairs Mitophagy

To confirm the regulation of AMPK on mitophagy and mitochondrial ATP production, expression of the two genes encoding catalytic subunits of AMPK (PRKAA1/2) was knocked down in BeWo cells and a stable cell line was established. The mRNA levels of *PRKAA1/2* were reduced by 2.03- and 2.07-fold (both *p* < 0.001), respectively, compared to control BeWo cells, while the protein levels in AKD cells were reduced by 2.5-fold compared to control BeWo cells (*p* < 0.001; Supplementary Fig. 2).

**Fig. 4.**
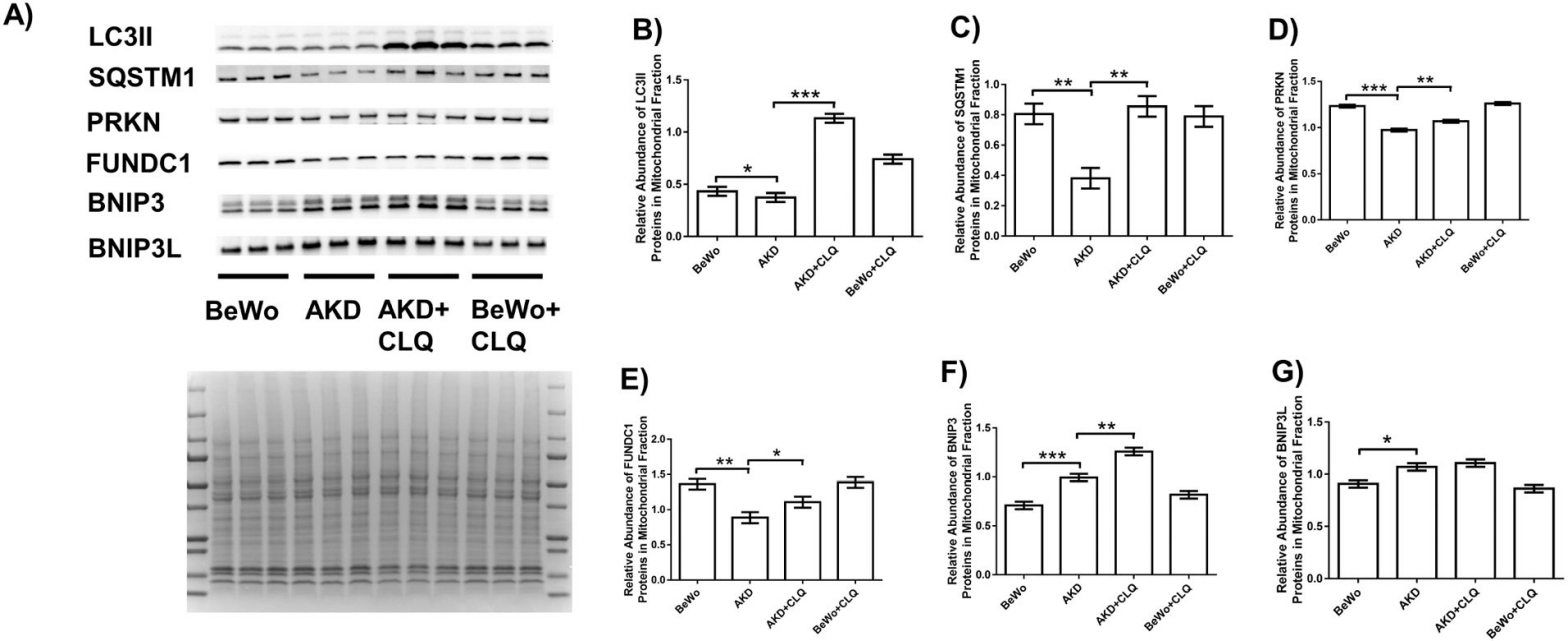
Reduced autophagy flux and mitophagy mediators by PRKAA1/2 knockdown in human trophoblast cell line BeWo. (A) Western blotting analysis of LC3II, SQSTM1, PRKN, FUNDC1, BNIP3, and BNIP3L proteins in mitochondrial fractions in AKD and BeWo cells treated with CLQ or without treatment and MemCode staining of the blot. Quantification of the relative abundance of LC3II (B), SQSTM1 (C), PRKN (D), FUNDC1 (E), BNIP3 (F), and BNIP3L (G) proteins by normalizing density of a band in Western blot to MemCode staining signal in the entire lane. Data are presented as the mean ± SEM (n = 3); * *p* < 0.05; ** *p* < 0.01; *** *p* < 0.001.

The abundance of LC3II proteins in mitochondrial fractions was reduced by 1.16-fold (*p* < 0.05) in AKD compared to control BeWo cells and increased by 3.03-fold (*p* < 0.001) by CLQ (Fig. 4B). The mitophagy marker SQSTM1 demonstrated a similar pattern of changes. The abundance of SQSTM1 protein was reduced by 2.11-fold (*p* < 0.01) in AKD compared to control BeWo cells and increased by 3.03-fold (*p* < 0.01) by CLQ (Fig. 4C). Among mitophagy mediators, the abundance of PRKN and FUNDC1 proteins in AKD cells was reduced by 1.27- (*p* < 0.001) and 1.54- (*p* < 0.01) fold, respectively, compared to control BeWo cells, and increased by 1.15- (*p* < 0.01) and 1.25- (*p* < 0.05) fold by CLQ, respectively (Fig. 4D,E). In contrast, the abundance of BNIP3 proteins in AKD cells was increasedby 1.40-fold (*p* < 0.001), compared to control BeWo cells, and increased by 1.26- (*p* < 0.01) fold by CLQ, respectively (Fig. 4F). The abundance of BNIP3L proteins in AKD cells was increased by 1.18-fold (*p* < 0.05) compared to control BeWo cells but was not altered by CLQ (Fig. 4G).

### 3.5 PRKAA1/2 Knockdown Elevated Mitochondrial Membrane Potential

**Fig. 5.**
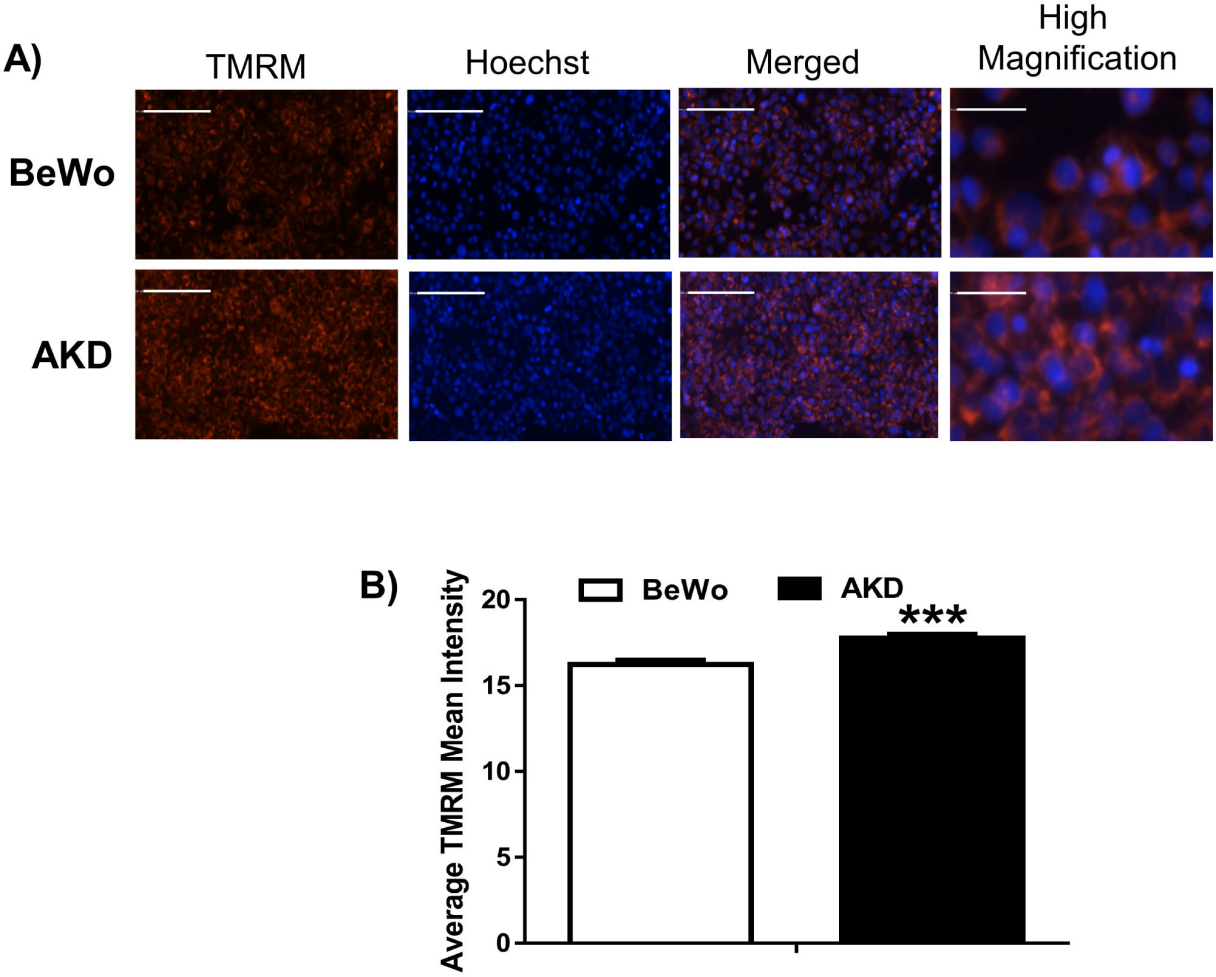
Mitochondrial membrane potentials increased by PRKAA1/2 knockdown in human trophoblast cell line BeWo. (A) Representative staining of TMRM (red) and Hoechst 33342 (blue) in PRKAA1/2 knockdown (AKD) and control BeWo cells (Bar = 200 *μ*m except in images in high magnification where bar is 500 *μ*m). (B) Average TMRM mean intensity normalized to the area of cells surface in each well containing 40,000 AKD or BeWo cells. Data are presented as the mean ± SEM (n = 6); *** *p* < 0.001.

Mitochondrial oxidative phosphorylation and ATP production are dependent on the finely tuned mitochondrial membrane potential [38]. To investigate the effect of AMPK knockdown on mitochondrial membrane potential, TMRM staining was conducted and compared between AKD and control BeWo cells. The average TMRM Mean intensity in AKD cells was increased by 1.094-fold (*p* < 0.001), compared to that in control BeWo cells, indicating elevated mitochondrial membrane potential after knockdown of *PRKAA1/2* genes (Fig. 5A,B).

### 3.6 PRKAA1/2 Knockdown Reduced Mitochondrial ATP Production

**Fig. 6.**
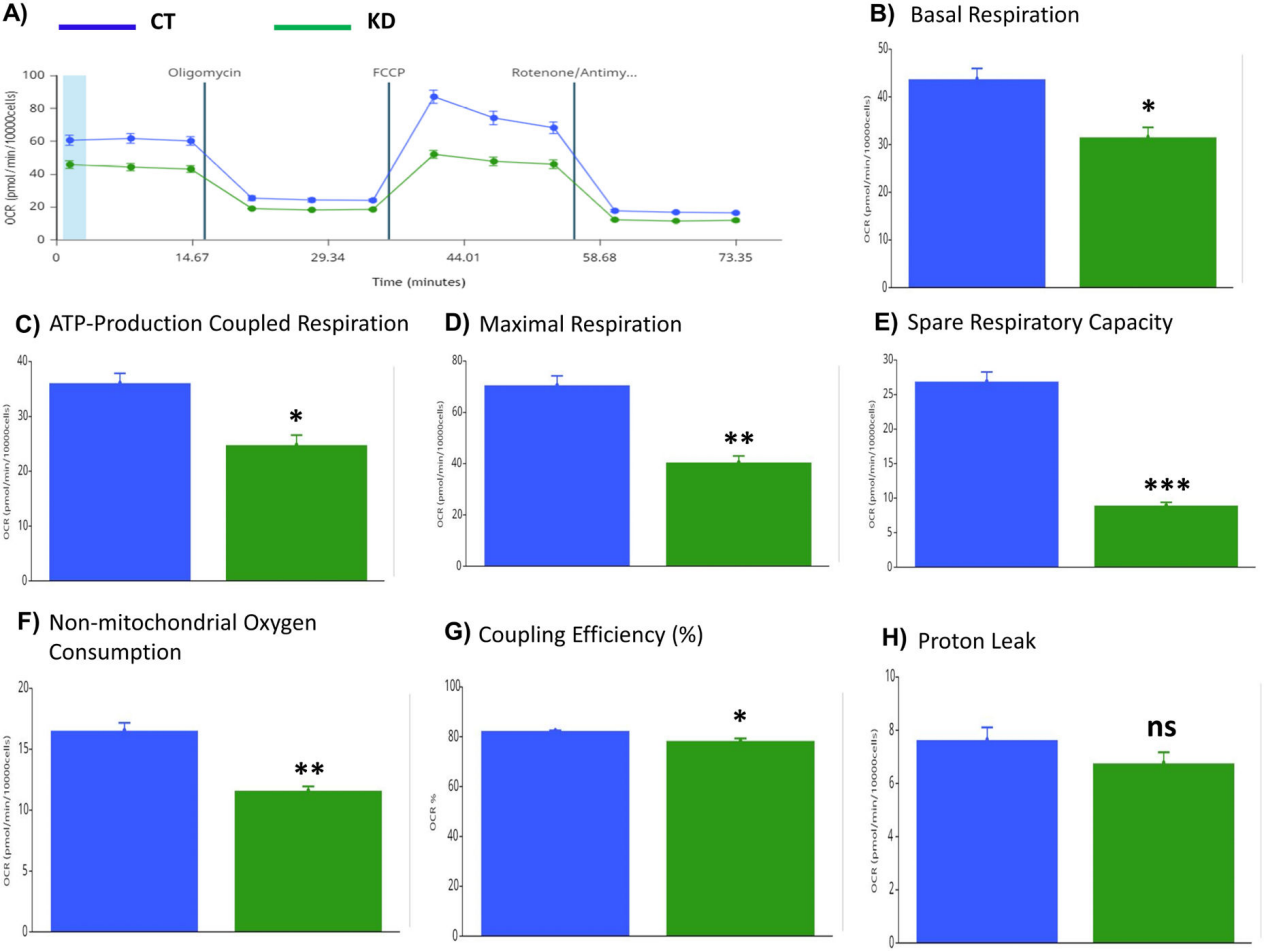
Reduced mitochondrial ATP production by PRKAA1/2 knockdown in human trophoblast cell line BeWo. (A) Dynamic oxygen consumption rate (OCR) in PRKAA1/2 knockdown (KD) and control (CT) cells in the presence of Oligomycin A, FCCP, Rotenone/Antimycin measured by Seahorse Mito Cell Stress Test. (B–H) Comparison of OCR for the basal respiration (B), maximal respiration (C), spare respiratory capacity (D), ATP production-coupled respiration (E), and non-mitochondrial oxygen consumption (F), and proton leak (H) between KD and CT cells. Data are presented as the mean ± SEM (n = 3); * *p* < 0.05; ** *p* < 0.01; *** *p* < 0.001; ns, not significant.

To investigate whether impaired mitophagy and elevated mitochondrial membrane potential affect mitochondrial ATP production, Seahorse cell mito stress test was conducted on AKD and control BeWo cells. ATP production via mitochondrial oxidative phosphorylation was lower in basal respiration and in presence of oligomycin, FCCP, and rotenone/antimycin in AKD compared to CT cells (Fig. 6A). The basal respiration (Fig. 6B), maximal respiration (Fig. 6C), spare respiratory capacity (Fig. 6D), ATP production-coupled respiration (Fig. 6E), non-mitochondrial oxygen consumption (Fig. 6F) and coupling efficiency (Fig. 6G) were reduced by 1.54- (*p* < 0.05), 1.74- (*p* < 0.05), 3- (*p* < 0.01), 1.46- (*p* < 0.001), 1.42- (*p* < 0.01), and 1.05- (*p* < 0.05) fold, respectively, in AKD compared to CT cells, while there was no difference in proton leak between these two cell types (Fig. 6H).

## 4. Discussion

Mitophagy plays a critical role in maintaining mitochondrial homeostasis in response to nutritional stresses, primarily monitored by the cell energy sensor AMPK and associated signaling. During human pregnancy, placental mitochondria are challenged by potent oxidative and nitrative stresses and many other deleterious factors [39,40]. Thus, effective mitophagy is critical for maintaining proper mitochondrial hemostasis and functions. To date, accumulating evidence supports that major pregnancy related disorders are associated with altered mitochondrial functions and/or autophagy [28,41-47], but mitophagy pathways and the role of AMPK signaling in the regulation of mitophagy remain unclear in human trophoblast cells. Our study for the first time delineated major mitophagy pathways and confirmed that AMPK signaling stimulates mitophagy in BeWo human trophoblast cells via PRKN and FUNDC1 mediated pathways (Figs. 2,3). Lower AMPK protein abundance reduces mitophagy and mitochondrial ATP production (Figs. 4,6), coincident with elevated mitochondrial membrane potential (Fig. 5).

We for the first time elucidate the regulatory effect of AMPK signaling in human trophoblast cells by analyzing three major mitophagy pathways by AMPK overactivation and AMPK knockdown. The major mitophagy pathways mediated by PINK1/PRKN, BNIP3/3L and FUNDC1 have been characterized in other cell types including cardiomyocytes and neurons [13,18], reflecting a rescue strategy to promote cell survival in response to a variety of stresses [48,49]. Our study found these mitophagy pathways are present in human trophoblast cells (Figs. 3,4). More importantly, for all mitochondrial receptors or mitophagy mediators (LC3II, SQSTM1, PRKN, BNIP3/BNIP3L and FUNDC1) investigated in this study, the pattern of changes in response to AMPK activation is opposite to that in response to AMPK knockdown (Figs. 3,4). Thus, AMPK signaling, interweaved in complicated cellular communications, plays a critical role in the regulation of mitophagy. Conversely, the pattern of changes in BNIP3/BNIP3L is opposite to that of PRKN and FUNDC1 and negatively corelated with mitophagy. Enhanced mitophagy in response to AMPK activation is coincident with reduced BNIP3/3L protein abundance in mitochondrial fractions (Fig. 3) while impaired mitophagy in response to AMPK knockdown is coincident with increased BNIP3/3L protein abundance in mitochondrial fractions (Fig. 4). These observations suggest that AMPK may regulate mitophagy via several pathways (PRKN, FUNDC1) and BNIP3/3L mediated mitophagy may serve as a counterregulatory mechanism in the regulation of mitophagy. Trophoblast cells are metabolically active and imposed many stresses such as nutritional, oxidative, nitrative and hypoxia stresses [3,50], thus requiring different mitophagy pathways to respond to multiple stress conditions. How these different pathways are activated in the placenta remains unclear; our study indicates that AMPK is a key regulator.

AMPK knockdown in human trophoblast cells decreases mitochondrial ATP production, possibly via hyperpolarization of mitochondrial inner membrane and impaired mitophagy. Mitochondrial membrane potential is critical for mitochondrial ATP production and the membrane potential must be kept in a narrow range to maintain sustainable ATP production [38]. However, unlike in excitable cells including cardiomyocytes and neutrons, how mitochondrial membrane potential is controlled in human trophoblast cells has not been reported, to the best of our knowledge. Our study found that AMPK knockdown reduces mitochondrial ATP production (Fig. 6A), which was similar to that found in the mouse trophoblast stem cell line SM-10 [51]. In addition to reduced basal ATP production, the underlying mechanisms responsible for reduced mitochondrial ATP production in AKD cells include reduced maximal respiration (Fig. 6C), spare respiratory capacity (Fig. 6D), and ATP production-coupled respiration (Fig. 6E), indicating that AMPK knockdown exerts broad effects on electron transport chain complexes and ATPase activities. Interestingly, our study demonstrated that mitochondrial membrane potential in cells with PRKAA1/2 knockdown was increased 10% (Fig. 5) while mitochondrial ATP production was reduced 32% in basal levels (Fig. 6). These observations indicate that mitochondrial membrane potential contributes largely to mitochondrial ATP production in human trophoblast cells, and therefore, mitochondrial membrane potential must be controlled in a narrower range to optimize mitochondrial ATP production compared to other cell types. Conversely, hyperpolarization of the mitochondrial inner membrane in response to AMPK knockdown may contribute to impaired mitophagy. It is known that depolarization of mitochondrial inner membrane, indicated by reduced mitochondrial membrane potential, is considered to be the trigger of mitophagy, especially that mediated by PINK1/PRKN pathway [52,53]. Therefore, the reduced PRKN protein levels in mitochondrial fractions in AKD cells, together with reduced LC3II and SQSTM1 (Fig. 4), support that mitophagy and mitochondrial membrane potential are orchestrated in response to AMPK signaling.

There are several weaknesses in our study due to unavoidable limitations. First, we could not include an AMPK antagonist as a negative control for AICAR induced AMPK activation because there is no reliable AMPK antagonist available [54]. Dorsomorphin, also called Compound C, has been widely used as an AMPK antagonist [55] but its inhibition of AMPK is nonspecific [56,57] and more than 50 other protein kinases are inhibited simultaneously [54]. However, our AMPK knockdown strategy confirms the regulation of AMPK on mitophagy and mitochondrial function in an inhibitory context (Figs. 4,6). Second, human primary trophoblast cells do not proliferate in culture, so we used BeWo cells that are widely applied in mechanistic studies on placentas and trophoblast cells [58]. However, as a cancer cell line [59], BeWo cells might have acquired additional features that affect mitophagy and mitochondrial functions. Thus, some findings from BeWo cells should be validated in human primary cytotrophoblast cells. Third, in the mitophagy flux assay, the accumulation of FUNDC1 and BNIP3 in mitochondrial fractions was similar in response to the combination of AICAR and CLQ and CLQ alone (Fig. 3C,D), possibly because mitophagy receptors are inactive under resting conditions, and their activities are elicited differently upon signals triggering mitochondrial damage [16]. While Western blotting analysis of mitophagy receptors is the most reliable to study autophagy/mitophagy [55], other complementary methods may be included in future validation studies of some findings in this study.

To date, the dynamic changes of placental mitophagy during pregnancy and the cause-effect relationship of placental mitophagy and pregnancy related disorders remain unclear, but a handful of studies have demonstrated the link between autophagy/mitophagy defects in placental tissue or trophoblast cells in major pregnancy related disorders such as gestational diabetes mellitus and preeclampsia [28,41-47]. However, due to technical limitations with *in vivo* studies and the lack of reliable animal model for mechanistic studies, mitophagy in the placenta has remained poorly understood. While characterizing mitophagy in human trophoblast cells, we are currently investigating the cause of altered placental mitophagy using primary trophoblast cells or tissues from women with major pregnancy related disorders, aiming to dig out the underlying mechanisms present in the placental-fetal unit during pregnancy. Thus, this study provides a conceptual foundation to conduct future mechanistic studies on placental mitophagy in normal pregnancy and pathophysiological status.

## 5. Conclusions

This study indicates that major mitophagy pathways mediated by PRKN, FUNDC1, BNIP3/BNIP3L are present in human trophoblast cells and AMPK signaling regulates mitophagy via PRKN and FUNDC1 mediated mitophagy pathways, which may affect mitochondrial membrane potential and mitochondrial ATP production.

## Supplementary Material

Supplementary Figure 1

Supplementary Figure 2

## Figures and Tables

**Table 1.** Primary antibodies applied in Western blotting analysis.

Name	Company	Cat. No.	Species	Dilution
total AMPK	Cell Signaling	2532	rabbit	1:1000
p-AMPK (Thr172)	Cell Signaling	2535	rabbit	1:1000
total ACC	Cell Signaling	3676	rabbit	1:1000
p-ACC (Ser79)	Cell Signaling	11818	rabbit	1:1000
total ULK1	Cell Signaling	8054	rabbit	1:1000
p-ULK1 (Ser555)	Cell Signaling	5869	rabbit	1:1000
p-ULK1(Ser757)	Cell Signaling	14202	rabbit	1:1000
LC3	Cell Signaling	2775	rabbit	1:5000
SQSTM1	Cell Signaling	8025	rabbit	1:1000
PRKN	Abclonal	A0968	rabbit	1:1000
FUNDC1	Abclonal	A16318	rabbit	1:1000
BNIP3	Cell Signaling	44060	rabbit	1:5000
BNIP3L	Cell Signaling	12396	rabbit	1:5000
ACTB	Cell Signaling	3700	mouse	1:5000

## Abbreviations

AICAR: 5-Aminoimidazole-4-carboxamide ribonucleotide
ACC: Acetyl-CoA carboxylase
AMPK: 5’ adenosine monophosphate-activated protein kinase
BNIP3: BCL2/adenovirus E1B 19 kDa protein-interacting protein 3
BNIP3L: BCL2 Interacting Protein 3 Like
FUNDC1: FUN14 domain containing 1
LC3II: Microtubule-associated proteins 1A/1B light chain 3B
MFN2: Mitofusin-2
PINK1: PTEN-induced kinase 1
PRKN: Parkin
SQSTM1: Sequestosome 1
ULK1: Unc-51 like autophagy activating kinase 1
