# Supplementary figures and images for: AMPK Signaling Regulates Mitophagy and Mitochondrial ATP Production in Human Trophoblast Cell Line BeWo
Source: Front Biosci (Landmark Ed). Author manuscript; Available in PMC 2023 Jan 10. (PMC9830999; doi:10.31083/j.fbl2704118)

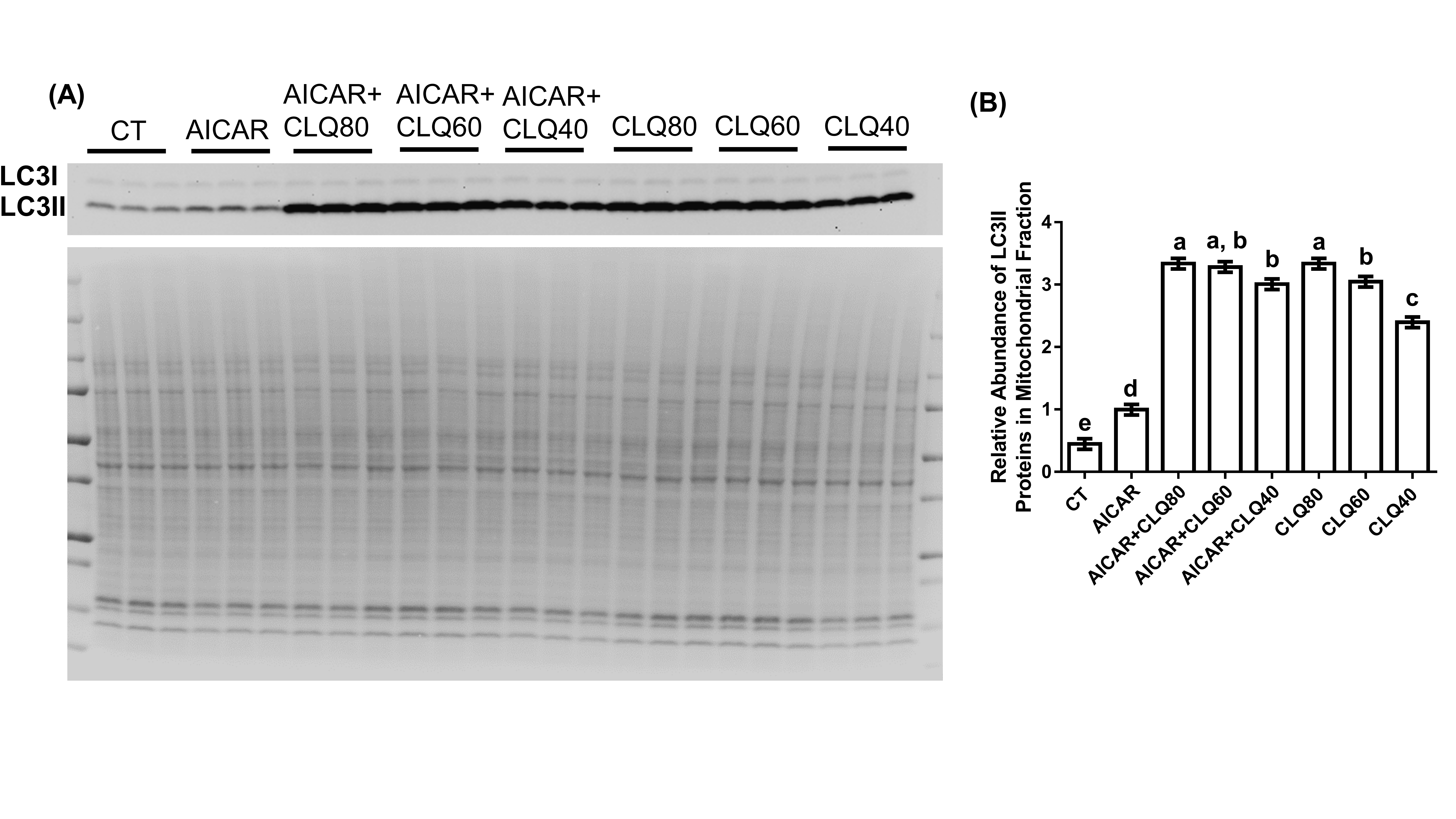

Supplement: Supplementary Figure 1 [file NIHMS1858666-supplement-Supplementary_Figure_1.tif]

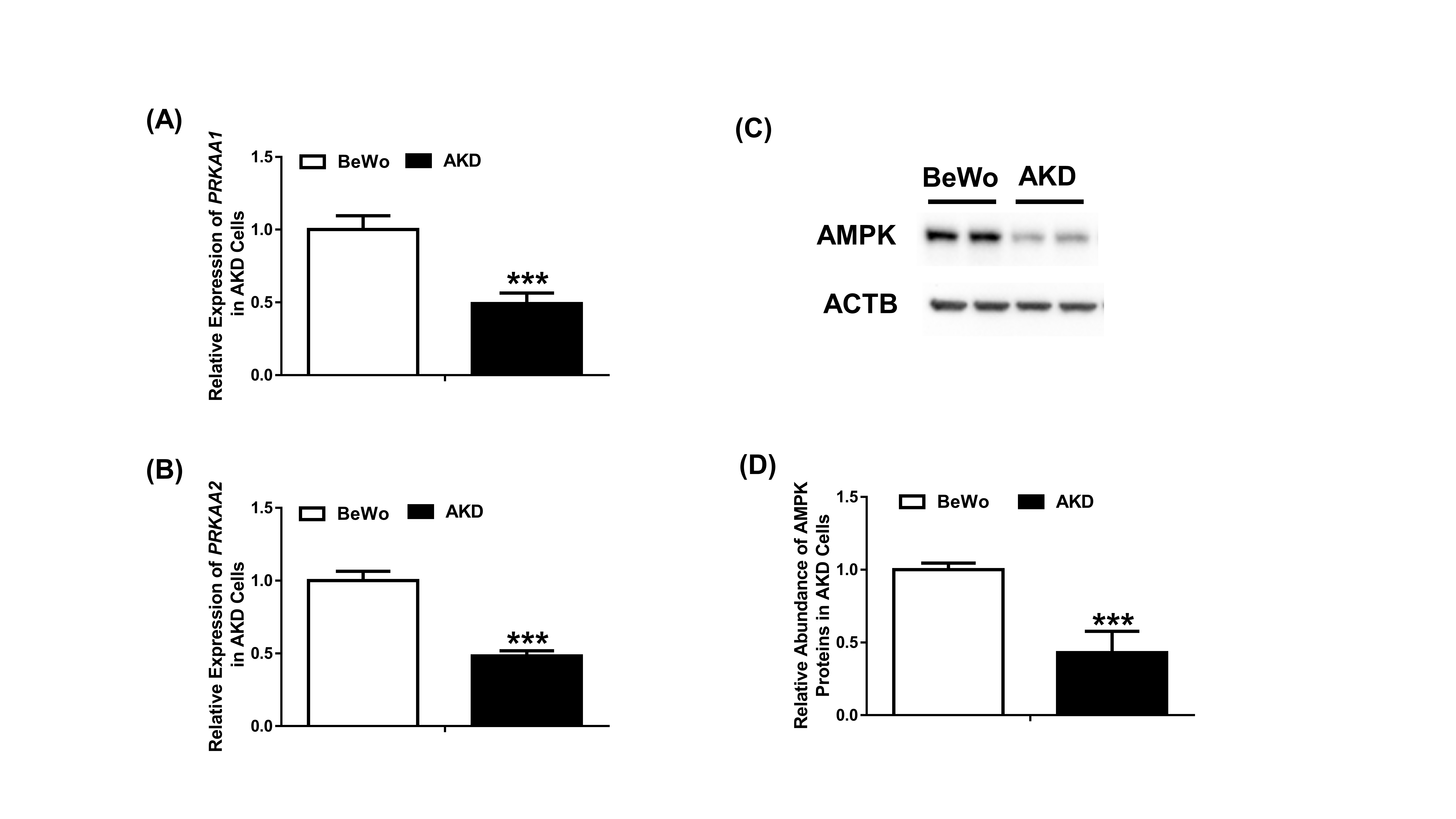

Supplement: Supplementary Figure 2 [file NIHMS1858666-supplement-Supplementary_Figure_2.tif]
